# Evolution of the Microstructure and Phase Composition of the Products Formed in the Reaction between Iridium and W_2_B

**DOI:** 10.3390/ma15217522

**Published:** 2022-10-26

**Authors:** Denis A. Bannykh, Victor V. Lozanov, Tatyana A. Gavrilova, Anatoly I. Beskrovny, Natalya I. Baklanova

**Affiliations:** 1 Institute of Solid State Chemistry and Mechanochemistry SB RAS, 18 Kutateladze Str., Novosibirsk 630090, Russia; 2 Rzhanov Institute of Semiconductor Physics SB RAS, 13 Lavrentiev Ave., Novosibirsk 630090, Russia; 3 Joint Institute for Nuclear Research, 6 Joliot-Curie Str., Dubna 141980, Russia

**Keywords:** tungsten, iridium, ternary boride, microhardness, intermetallics, microstructure

## Abstract

In the present study, we perform a systematic examination of the products formed by mixing and heating of tungsten boride and iridium powders at different ratios in a broad temperature range using qualitative and quantitative X-ray analysis and time-of-flight neutron diffraction (TOF-ND), in combination with scanning electron microscopy/energy-dispersive spectroscopy (SEM/EDS) performed at different accelerating voltages. The well-known and unknown ternary W–Ir–B phases were detected. The Vickers microhardness value for the new ternary W_2_Ir_5_B_2_ boride was measured. Based on these findings, the ternary W_2_Ir_5_B_2_ boride can be considered hard.

## 1. Introduction

Tungsten–iridium- and tungsten–iridium–boron-based materials are commonly demanded in the industry. These materials can be used for fabricating engines, cutting tools, wear-resistant and antioxidant coatings, as well as electrocatalysts due to the outstanding properties of both metals, including high melting points (2447 °C for Ir and 3422 °C for W), high level of mechanical properties, high hardness, and good oxidation resistance [1,2,3,4,5,6,7,8,9,10,11]. There are numerous examples where the synergistic effect of both metals significantly improved the properties of designed materials. Thus, the addition of iridium to tungsten increased the yield strength of the W–Ir alloy by 75% at 1727 °C [12] and the ductility of tungsten at room temperature [13]. Therefore, iridium can be considered a real alternative to rhenium, which was previously used in superalloys [12,13]. Good oxidation- and ablation-resistant W–Ir coatings for carbon supports were developed and successfully tested at high temperatures in an oxidative atmosphere [1,2]. Another positive example of the synergistic effect of both metals is the development of low-temperature brazing alloys for joining tungsten for high-temperature service [14]. The iridium–boron alloy systems with various proportions of tungsten have been used for this purpose. The addition of tungsten to the Ir–B alloy was found to increase the remelt temperature for joining. The remelt temperature for joining of tungsten varies in the range of 1800–2120 °C depending on the alloy composition. It suggests that the ternary W–Ir–B compounds with high melting points could be formed during joining.

The active expansion of the W–Ir and especially the W–Ir–B-based materials to new application areas is impeded by the fact that the available data on their composition, structure, and properties are scarce and contradictory. Several intermetallic compounds are known to exist in the Ir–W system, including W_3_Ir (P 4_2_/mnm), W_x_Ir_1−x_ solid solution with a very wide homogeneity range (from 23–25 at.% to 52–57 at.% W) (P 6_3_/mmc), as well as two ordered WIr (P mma) and WIr_3_ (P 6_3_/mmc) phases, each of them also having a homogeneity range of ~2 at.% W and 6 at.% W, respectively [15,16,17,18,19,20,21]. Both ordered phases are displayed within the homogeneity region of W_x_Ir_1−x_ solid solution. This might cause significant difficulties for interpreting the research results.

According to phase equilibria in the W–Ir–B system, there are only two ternary W_2_Ir_3_B_6−x_ (x ≈ 1) [22] and W_l8_Ir_l3_B_69_ phases [23,24] in the boron-rich portion of the W–Ir–B system. In the metal-rich portion, the rapidly solidified W–Ir–B alloy (the 60:20:20 atomic ratio) was found to be composed of two crystalline phases (namely, the orthorhombic IrW and tetragonal W_2_B phases); no ternary borides were found [3]. Nevertheless, taking into account the high diversity of ternary borides for similar systems (Mo–Ir–B [22,25,26], Hf–Ir–B [27,28,29,30], and Zr–Ir–B [28,31]), one can suggest that ternary borides other than the aforementioned one could be formed in the W–Ir–B system. Here, it should be emphasized that the simultaneous presence of very heavy (W, Ir) and very light (B) elements in the composition is the main difficulty in investigating such a system.

In the present study, we performed a systematic examination of the products formed by mixing and heating of tungsten boride and iridium powders at different ratios in a broad temperature range using qualitative and quantitative X-ray analysis and time-of-flight neutron diffraction (TOF-ND), in combination with scanning electron microscopy/energy-dispersive spectroscopy (SEM/EDS) performed at different accelerating voltages. The well-known and unknown ternary W–Ir–B phases were detected. The Vickers microhardness value for the new ternary W_2_Ir_5_B_2_ boride was measured. Based on these findings, ternary W_2_Ir_5_B_2_ boride can be considered hard. Our findings expand the class of hard materials.

## 2. Materials and Methods

### 2.1. Initial Substances

Iridium powder (purity ≥ 99.96%, GOST standard 12338–81, Krasnoyarsk, Russia) with the particle size D50 ~22 μm (Microsizer 201A analyzer) was mechanically pre-treated in a PM 100 CM planetary ball mill (Retsch, Haan, Germany) according to the procedure described by Bannykh et al. [32]. The lining of vials and the grinding media were made of WC. The particle size composition of powders was measured using a Microsizer 201 particle analyzer (Russian Federation State Standard GOST 57923-2017). The particle size of the mechanically treated iridium powder was equal to D_50_~7.4 μm, D_90_ = 15 μm. The habitus of particles was plate-like with a thickness of 100–300 nm. According to the EDS analysis, the iridium powder contained tungsten as a contaminant (~0.5 wt.%), which can be explained by the use of lining and grinding media composed of tungsten carbide.

The initial tungsten boride (Ltd. “Yumax”, Ufa, Russia) contained 16 wt.% of the WB phase in addition to the dominant W_2_B phase. The initial tungsten boride was mechanically treated in a planetary ball mill. The particle size was found to be equal to D_50_ ≈ 400 nm. After mechanical treatment, WC (1–2 wt.%) was detected as a contaminant. The cell parameters (a = 5.569 Å, c = 4.744 Å) and cell volume (V = 147.0 Å^3^) of the W_2_B phase were in good agreement with the reference data (ICSD 42528) reported by Havinga et al. [33] and slightly lower than those reported by Kiessling [34,35]. This fact confirms the near-stoichiometric composition of W_2_B. Contrariwise, the cell volume of the WB phase was estimated to be 162.4 Å^3^, which corresponds to the composition of WB_0.96_ [34].

### 2.2. Preparing the W–Ir–B Products and Their Characterization

The W–Ir–B-based materials were obtained by mixing tungsten boride and iridium powders at different ratios in an agate mortar followed by consolidation by uniaxial pressing (P = 5 MPa, 30 s) at ambient temperature. The molar ratios of tungsten boride and iridium powder are listed in Table 1.

Next, the pressed powder mixtures were loaded into grafoil boxes and placed into a high-temperature SNVE 1,7.3.1,7/20 vacuum furnace (Prizma, Iskitim, Russia), evacuated to P~1.33∙10^−3^ Pa, heated to a given temperature (1000–1600 °C, with an increment of 100 °C) at a rate of 640 °C/h, and exposed to the given temperature for 1 h. The samples were cooled down under continuous vacuuming at a rate of 300 °C/h (5 °C/min).

The phase composition was studied using a Bruker D8 Advance X-ray powder diffractometer (Bruker Corporation, Billerica, MA, USA) employing CuKα radiation in the 10° ≤ 2Θ ≤ 110° range. The time-of-flight neutron diffraction (TOF-ND) patterns of the product obtained after heating the powdered 1:1 mixture (1600 °C, 4 h) were collected using a real-time diffractometer of the IBR-2 reactor (FLNP JINR, Dubna, Russia) [36]. The surface morphology and local elemental composition of the samples were analyzed by scanning electron microscopy coupled with energy dispersive spectroscopy at an accelerating voltage of 15 kV, 20 kV, and 6 kV. The experimental details of analytical techniques are presented in the Supplementary Materials.

In order to measure the microhardness of different phases, the powdered products were packed into epoxy resin and polished. Vickers microhardness measurements were performed using a DuraScan-50 hardness testing machine (EMCO-TEST, Kuchl, Austria) at a load of 0.245 N (25 gf) in accordance with the ASTM E384-17 standard test method. The dwell time was 10 s.

## 3. Results and Discussion

### 3.1. Characterization of the Products Obtained via the Reaction between Tungsten Boride and Iridium Powders in the 1000–1200 °C Temperature Range

**Figure 1:**
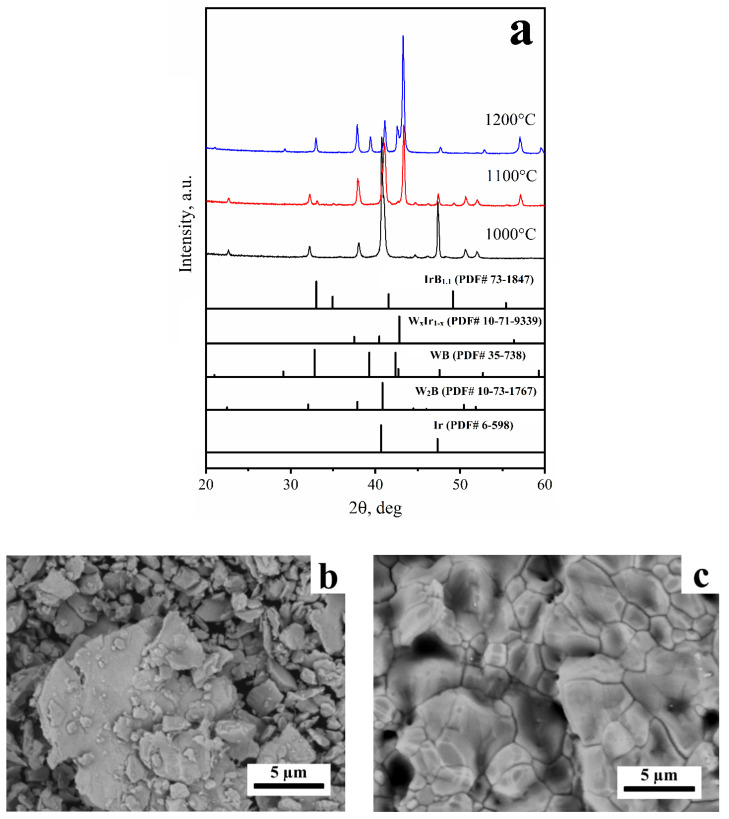
The XRD patterns (**a**) and SEM images ((**b**)—1100 °C, (**c**)—1200 °C) of the products obtained via the reaction between iridium and tungsten (1:1 mixture).

According to the phase equilibria in the binary Ir–B, W–B, and W–Ir systems, the lowest-melting eutectic was formed in the Ir–B system at 1248 °C [37]. Therefore, one can consider the reaction between iridium and tungsten boride in the 1000–1200 °C temperature range to be a solid-state reaction. The X-ray diffraction data for the heat-treated 1:1 mixtures at 1000–1200 °C are presented in Figure 1a. One can see that no additional peaks besides those belonging to the initial reagents were observed in the products obtained at 1000 °C. The first feature of the reaction between W_2_B and Ir is the emergence of the XRD peaks belonging to iridium boride, IrB_1.1_, and W_x_Ir_1−x_ intermetallics (2Θ = 57.05°) in the products obtained at 1100 °C. The composition of the intermetallic phase was calculated to be W_0.23_Ir_0.77_ in accordance with the Vegard’s rule. For calculation, the reference data by Tylkina et al. [18] and Raub et al. [15] were used. The formation of these products probably occurred according to reaction 1. Here, x is 0.23.

11xW_2_B + (22 − 12x)Ir → 10xIrB_1.1_ + 22W_x_Ir_1−x_ (1)

The phase composition of the products obtained at 1200 °C in the 1:1 mixtures was found to also be presented by WB and W_x_Ir_1−x_. The quantitative X-ray analysis showed that the content of the WB phase was 27 wt.% (1200 °C), which is much higher than that in the initial reagent (see Supplementary Materials). The additional quantity of the WB phase could have arisen from the reaction between iridium and the W_2_B phase. The peak maxima of the WB phase shifted noticeably towards large angles. This is accompanied by an increase in the WB cell volume from 162.4 Å^3^ to 163.4 Å^3^. Taking into account the cell volume–composition relationship for WB_1−x_ proposed by Kiessling [34], the phase composition of the WB_1−x_ phase was calculated to change from WB_0.96_ to WB_0.98_. The other phase formed via the reaction between iridium and tungsten boride at 1200 °C; the W_x_Ir_1−x_ substitutional solid solution (~73 wt.%) has a very broad homogeneous range, namely, x = 0.25–0.53.

**Figure 2:**
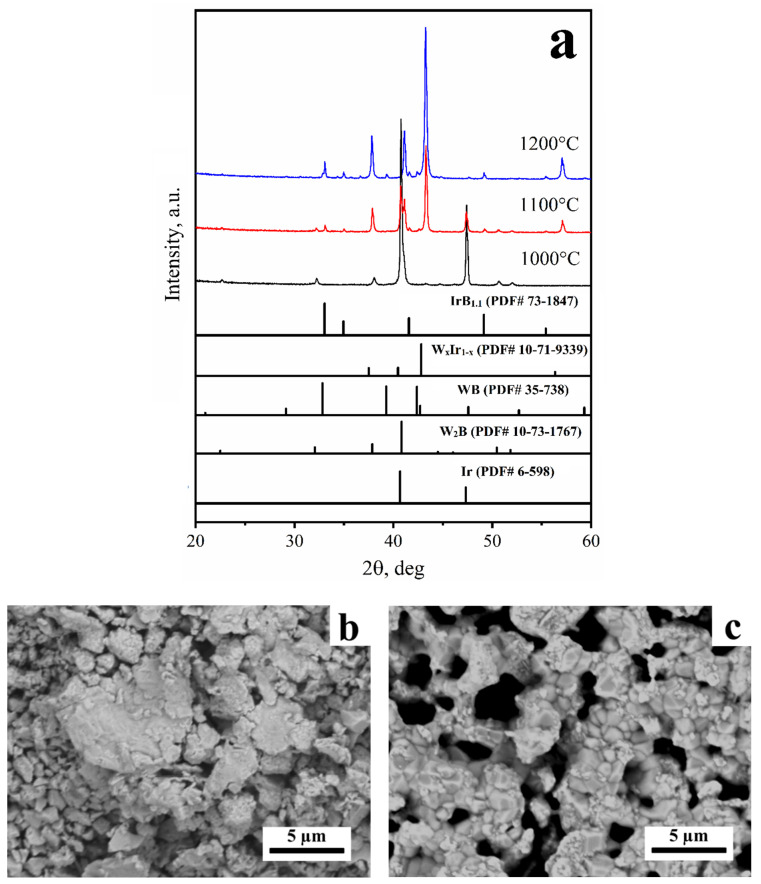
The XRD patterns (**a**) and SEM images ((**b**)—1100 °C, (**c**)—1200 °C) of the products obtained via the reaction between iridium and tungsten (3:1 mixture).

The behavior of the 3:1 mixture differs from that for the 1:1 mixture. After heat treatment at 1200 °C, the XRD peaks belonging not only to W_0.23_Ir_0.77_ (87 wt.%) and WB (4.5 wt.%), but also to IrB_1.1_ are observed (Figure 2a). The formation of the aforementioned products can be attributed to Reaction (2):

xW_2_B + (1 − x)Ir = xWB + W_x_Ir_1−x_ (2)

The morphology of the products obtained at 1200 °C was changed compared with that observed for the products at 1000 °C and 1100 °C; particles of irregular habitus were detected, being connected with each other by bridges (Figure 2b,c).

### 3.2. Characterization of the Products Obtained via the Reaction between Tungsten Boride and Iridium in the 1300–1600 °C Temperature Range

#### 3.2.1. The 1:1 Mixtures

**Figure 3:**
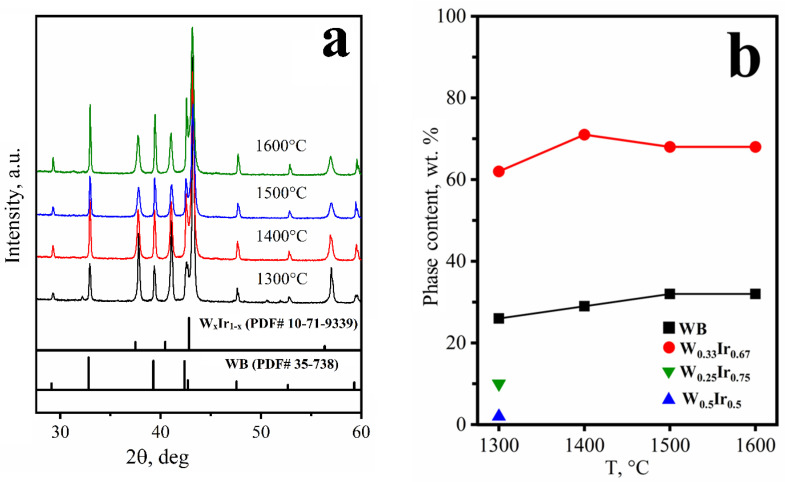
The XRD patterns (**a**) and phase composition (**b**) of the products depending on temperature.

According to the XRD analysis, only the WB and W_x_Ir_1−x_ phases are present in the products obtained in the 1300–1600 °C temperature range (reaction 2, Figure 3a). Broad asymmetric peaks with a complex contour were detected in the XRD pattern of the product formed at 1300 °C. These peaks can be fairly well described by three components corresponding to the compositions W_0.25_Ir_0.75_, W_0.5_Ir_0.5_, and W_0.33_Ir_0.67_. The latter one is dominant in accordance with the quantitative XRD analysis (Figure 3b). According to the data reported in [20], the ordered W_0.25_Ir_0.75_ (WIr_3_) and W_0.5_Ir_0.5_ (WIr) phases have crystal structure types MgCd_3_ and AuCd, respectively. The XRD peaks of the W_0.25_Ir_0.75_ and W_0.5_Ir_0.5_ intermetallic phases disappear as the temperature increases (1400 °C and above). This result is not surprising because the W_0.33_Ir_0.67_ phase is thermodynamically the most stable [38].

As mentioned above, tungsten boride was also found among the products obtained in the 1300–1600 °C temperature range. As the temperature rises, the composition of WB_x_ approaches WB_0.96_ [39]; furthermore, the coherent-scattering region increases gradually, which can argue in favor of the growth of WB grains. The results of quantitative XRD analysis are presented in Figure 3b. One can see that the W_x_Ir_1−x_:WB ratio is almost constant in the 1300–1600 °C temperature range.

**Figure 4:**
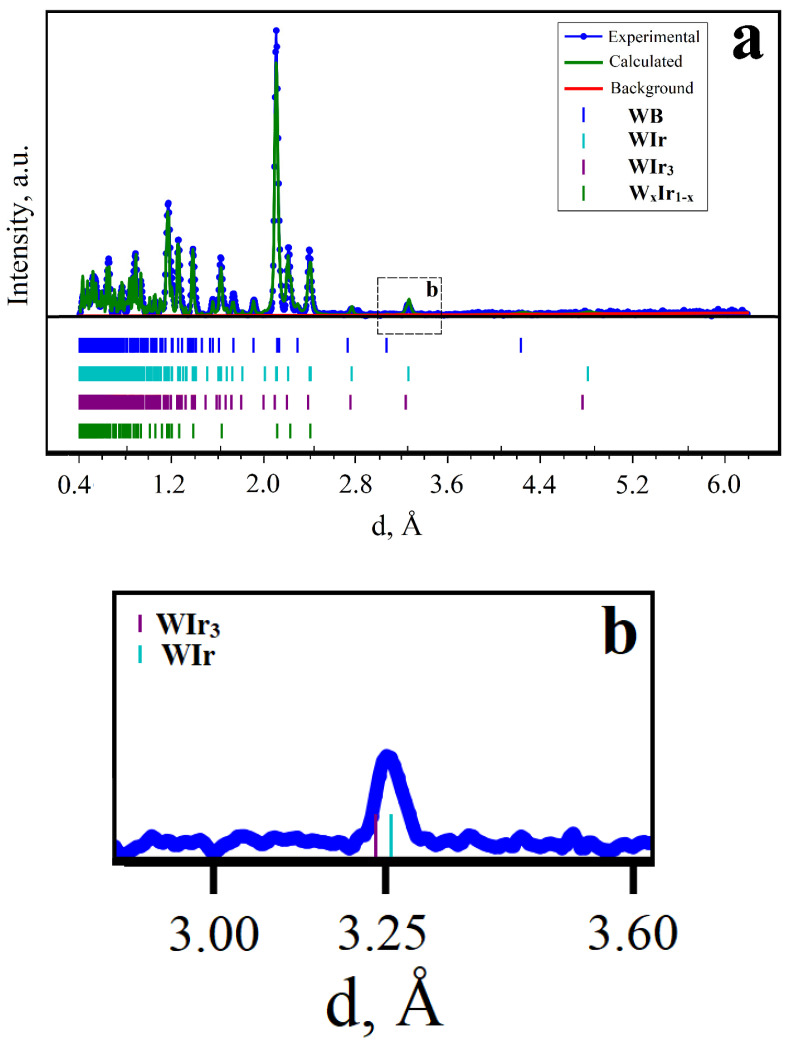
The TOF-ND pattern of the product obtained at 1600 °C, 4h (**a**). The TOF-ND superstructure peak for the ordered WIr_3_ and WIr phases (**b**).

Additional important information about the phase composition can be obtained from the TOF-ND analysis data (Figure 4). In addition to the peaks associated with the WB and W_x_Ir_1−x_ solid solution, low-intensity peaks at d~3.25 and 2.80 Å can be seen in the TOF-ND patterns (Figure 4a). Each of the broad peaks at d~3.25 Å and 2.80 Å can be deconvoluted into two components (Figure 4b). The comparison of peak positions allows us to ascribe them to the ordered intermetallic WIr and WIr_3_ phases [20]. The results of the quantitative TOF-ND analysis are summarized in Table 2. It follows from Table 2 that the intermetallic phase is made of ordered phases by 50%; the remaining part is the disordered W_x_Ir_1−x_ solid solution. The cell volume of WIr_3_ was 0.2% larger, and the cell volume of WIr was 0.8% smaller than those reported in the Springer Materials database. These results are in accordance with previously reported data about the homogeneity regions for the ordered intermetallic WIr and WIr_3_ phases [21].

Hence, the TOF-ND method allowed us to clearly detect the formation of both ordered and disordered intermetallic W–Ir phases. We would like to note that it could not be achieved by X-ray analysis because of the coincidence of high-intensity X-ray peaks of the ordered and disordered intermetallic phases and the absence of superstructure peaks belonging to the ordered WIr_3_ and WIr phases.

**Figure 5:**
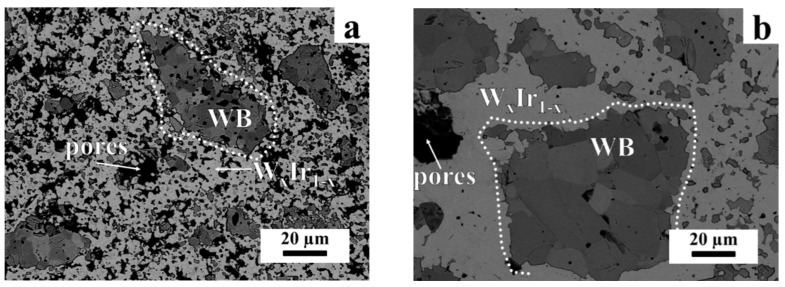

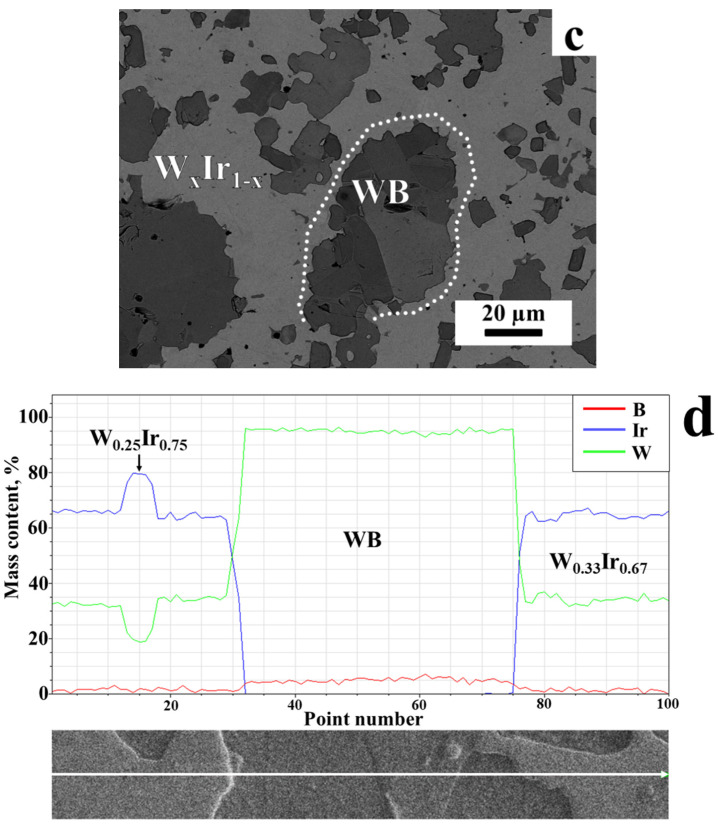
SEM images of the cross sections of the mixtures heat-treated at different temperatures: (**a**) 1400 °C; (**b**) 1500 °C; and (**c**) 1600 °C (accelerating voltage, 20 kV). (**d**)—The EDS analysis data (accelerating voltage, 6 kV) of the cross section of the product obtained at 1600 °C. Element distribution along the white arrow is shown.

Unfortunately, it was impossible to prepare a section of the sample heat-treated at 1300 °C to study its microstructure because the sample was characterized by high porosity, causing particle chipping during sample preparation (Supplementary Materials, Figure S1). Figure 5a–c show the typical SEM images of the cross-section of the products obtained at higher temperatures. The SEM/EDS analysis demonstrated that the grains varied in contrast, thus indicating that spots with different elemental compositions were formed. In accordance with the EDS analysis data, dark gray grains belonged to WB. The linear size of WB particles was found with the ImageJ software to be equal to 2–7 µm (T = 1400 °C), 5–17 µm (T = 1500 °C), and 6–20 µm (T = 1600 °C). As the temperature rose, the size of WB aggregates increased (70 µm, 100 µm, and 110 µm for 1400 °C, 1500 °C, and 1600 °C, respectively). The distinct contrast of an individual WB grain can be attributed to some deviation in composition and/or formation of a solid solution. WB aggregates are surrounded by the light gray W_x_Ir_1−x_ phase whose composition is represented by W_0.33_Ir_0.67_ (predominantly) and W_0.25_Ir_0.75_ (Figure 5d, respectively). The porosity decreases with increasing temperature. In general, the microstructures of the products obtained at 1500 and 1600 °C are almost indistinguishable. The phase compositions for both temperatures also coincide.

#### 3.2.2. The 3:1 Mixtures

**Figure 6:**
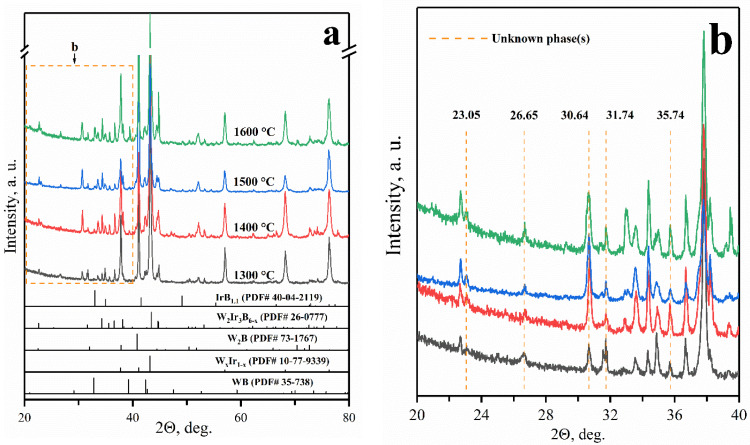
The XRD patterns of the Ir:W_2_B = 3:1 mixtures heat treated in the temperature range of 1300–1600 °C (**a**). Inset (**b**): the XRD peaks of an unknown phase(s).

The results of qualitative X-ray analysis of the 3:1 mixtures heated at different temperatures showed that the products are composed of the W_x_Ir_1−x_ (x ≈ 0.25), IrB_1.1_, WB, and W_2_Ir_3_B_6−x_ phases (Figure 6). The presence of the IrB_1.1_ phase in the product suggests that reactions involving liquids occur in the 1300–1600 °C temperature range. Another interesting feature is the emergence of XRD peaks belonging to the ternary W_2_Ir_3_B_6−x_ boride phase. According to the phase equilibrium in the W–Ir–B system, the W_2_Ir_3_B_6−x_ phase is a single ternary boride. Finally, the most important observation is the presence of a series of XRD peaks that cannot be ascribed to any known binary or ternary phases in the W–Ir–B system. These peaks are listed in Table S1 of the Supplementary Materials.

It follows from the TOF-ND pattern that the peaks belonging to IrB_1.1_, the intermetallic W_x_Ir_1−x_ solid solution, as well as the ordered WIr_3_ phase, have emerged. In addition, the non-identified peaks in the *d* = 0.4–1.0 Å range were detected. They can probably be related to the formation of an unknown ternary boride phase(s) (Supplementary Materials, Figure S2).

**Figure 7:**
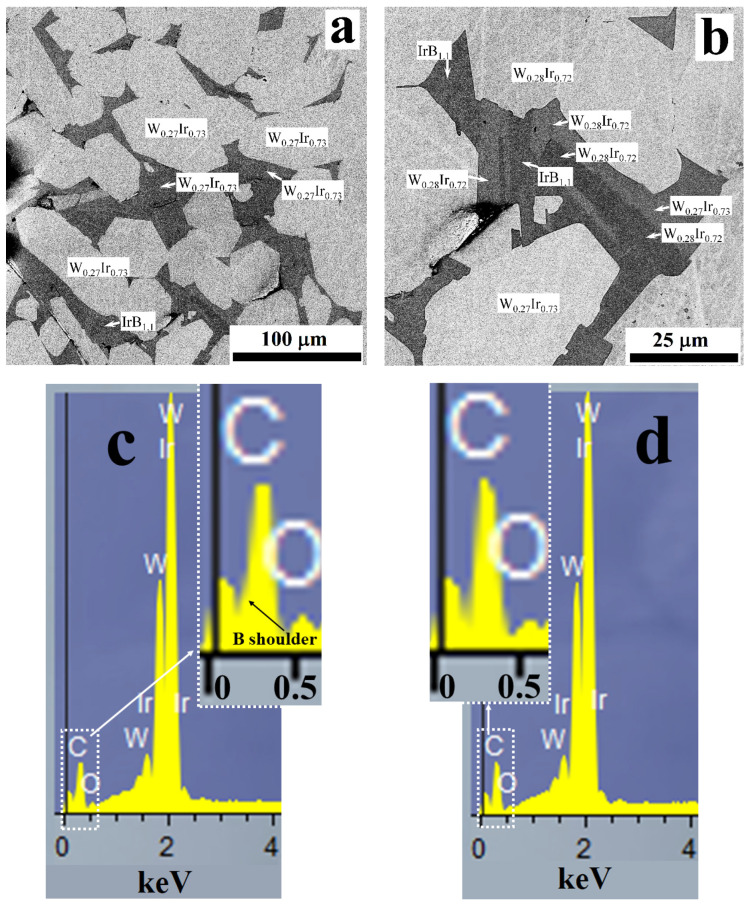
The BSE SEM/EDS images of two typical cross sections of the products obtained in the 3:1 mixtures (**a**,**b**). The EDS spectra taken from the selected areas: (**c**)—middle gray area belonging to the boron-containing phase; (**d**)—light gray area belonging to boron-free phase.

The BSE SEM/EDS images of two typical cross sections of the products obtained in the 3:1 mixtures are shown in Figure 7a,b (accelerating voltage of 20 kV). One can observe several types of areas distinct in color that indicate different elemental compositions. The dark gray areas were ascribed to IrB_1.1_. The EDS spectra were processed in terms of the W/Ir atomic ratio. Surprisingly, for both the light gray and middle gray large areas that clearly differ in contrast, the W:Ir ratio was almost similar and close to 0.27–0.28:0.73–0.72. This can imply that the contrast differed in these areas because of the presence of a third element (e.g., boron) in the middle gray areas. Indeed, a hardly noticeable shoulder with a maximum at ~180–200 eV is observed in the EDS spectrum taken from the middle gray area at an accelerating voltage of 20 kV (Figure 7c). One can note that the maximum coincides with the B-K line [40,41] and overlapped with the W-N (208, 212.2, 222.1, 229.5 eV) and Ir-N (234.8, 247 eV) lines [42]. In this special case, the M lines of tungsten and iridium are usually used to determine the elemental composition. As seen from Figure 7d, no shoulder was present in the EDS spectrum taken from the light gray area. It suggests that this area belongs to the boron-free phase.

**Figure 8:**
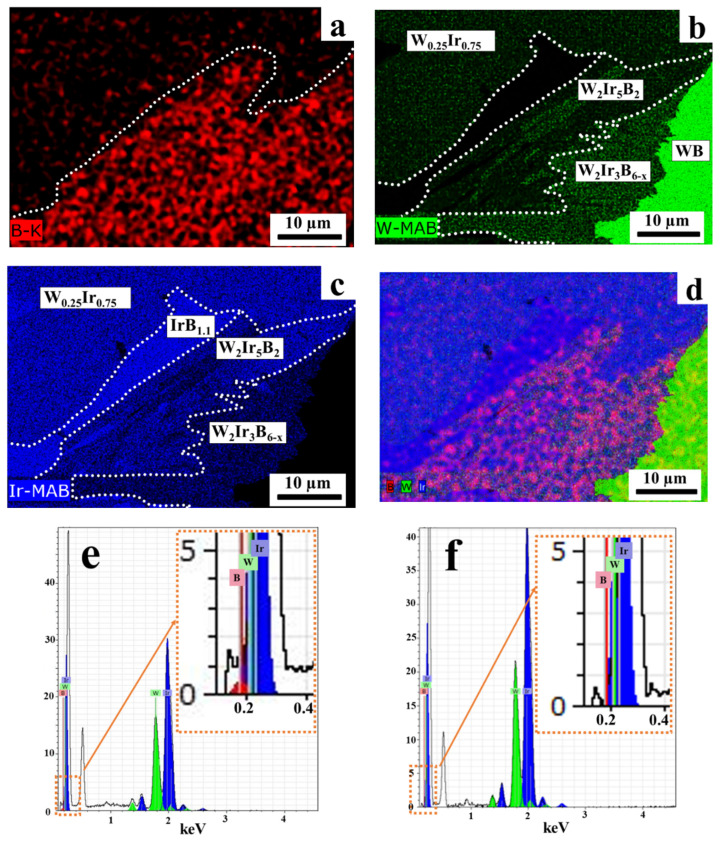
(**a**–**f**) The EDS spectra (accelerating voltage, 6 kV) of the cross sections of the products obtained at 1600 °C; (**a**–**c**) elemental mapping of random grains demonstrating the formation of different phases. Here, B, Ir, and W are colored by red, blue, and green, respectively; (**d**) cameo BS SEM/EDS image. The EDX spectra of the selected areas: W_2_Ir_5_B_2_ (**e**) and W_0.25_Ir_0.75_ (**f**) phases.

**Figure 9:**
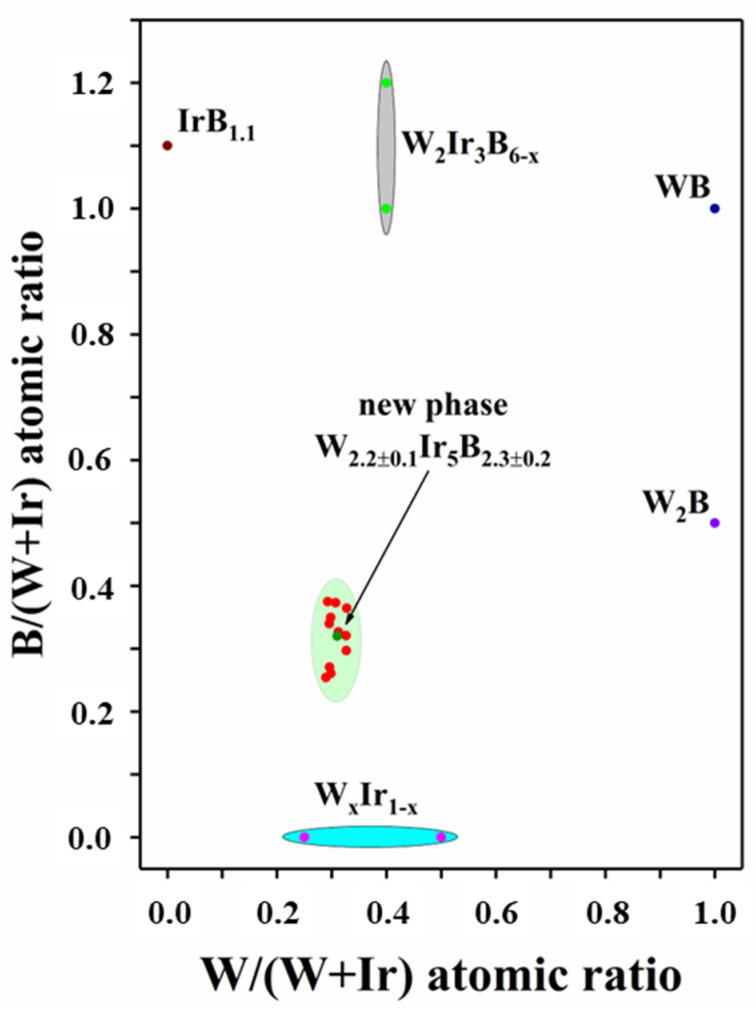
The B/(W + Ir) ratio as a function of the Ir/(W + Ir) ratio. The findings for the known phases of the binary W–Ir, B–Ir, W–B, and ternary W–Ir–B systems are also shown. Red points are the energy-dispersive X-ray spectrometry (EDS) data for W_2_Ir_5_B_2_; the reference data for the known compounds are as follows: wine-colored points—IrB_1.1_; royal blue points—WB; violet points—W_2_B; magenta points—W_x_Ir_1−x_; and green points—W_2_Ir_3_B_6−x_.

The EDS analysis performed at an accelerating voltage of 6 kV allowed us to determine the elemental composition more comprehensively (including detecting boron) (Figure 8). Elemental mapping of random grains shows that different phases are present. Together with the intermetallic W_0.25_Ir_0.75_ phase, another intermetallic phase, W_x_Ir_1−x_ (x ≈ 0.33), was also detected; however, it was a very rare occurrence (Supplementary Materials, Figures S3 and S4). In addition, the WB and IrB_1.1_ phases were also found. The most interesting fact is the presence of two types of middle gray areas simultaneously containing tungsten, iridium, and boron at different ratios. The EDX spectra of the selected areas are shown in Figure 8e,f. One of them had the W_2_Ir_3_B_6−x_ composition, where x = 0.35–0.87; the other one had a statistical composition W_2.2±0.1_Ir_5_B_2.3±0.2_ (Figure 8e). It is worth noting that the latter phase has never been previously reported for the W–Ir–B system. All the experimental EDS analysis data for new ternary phase in the present study were processed as the dependence of the B/(W + Ir) ratio on the Ir/(W + Ir) ratio (Figure 9). One should bear in mind that each detected ternary phase under consideration varies insignificantly in heavy metal contents, but can significantly vary in boron content. Thus, taking into account the EDS and XRD data, the W_2_Ir_5_B_2_ phase can be considered as a new ternary boride.

Thus, the data on phase and elemental compositions of the products formed by heat treatment of the 3:1 mixtures were obtained. The good reproducibility of the powder XRD, SEM/EDS, and TOF-ND analysis data unambiguously confirms that a novel ternary boride with tentative composition W_2_Ir_5_B_2_ is formed at temperatures higher than 1300 °C. No phase with a similar composition has been previously reported for the W–Ir–B system. In our opinion, a key factor contributing to the formation of ternary boride phases via the reaction between iridium and tungsten boride is the emergence of liquid iridium boride at temperatures above 1300 °C. This is a liquid phase, which promotes improved mass transfer and ensures the formation of ternary borides, W_2_Ir_3_B_6_ and W_2_Ir_5_B_2_.

### 3.3. Microhardness

**Figure 10:**
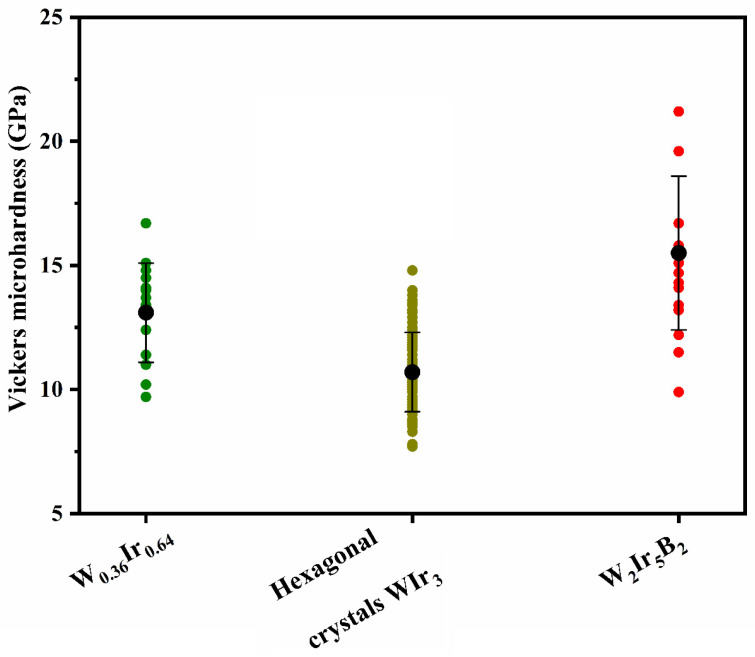
Microhardness of intermetallic areas and the new ternary W_2_Ir_5_B_2_ phase in the products obtained at 1600 °C, 4 h.

In this work, the Vickers microhardness values for binary intermetallic areas and new ternary W_2_Ir_5_B_2_ boride were measured at a load of 0.245 N. The results are presented in Figure 10. The mean microhardness of intermetallic areas (without boron) with composition W_0.36_Ir_0.64_ is ~13.1 ± 2.0 GPa (18 tests), whereas the microhardness of hexagonal WIr_3_ crystals was evaluated to be 10.7 ± 1.6 GPa (104 tests). These microhardness values are in good agreement with the data previously reported by Tylkina et al. for similar compositions [18] and are somewhat lower than those reported by E. Raub et al. [15] (Table 3). The microhardness value for the new ternary boride, W_2_Ir_5_B_2_, was measured for the first time and was equal to 15.5 ± 3.1 GPa (16 tests). Based on this result, the new ternary boride can be considered the hard phase. This value is lower than that reported previously for the ternary Hf–Ir–B boride of similar composition (18.2 ± 1.7 GPa for Hf_2_Ir_5_B_2_) [30].

## 4. Conclusions

The products of the reaction between iridium and tungsten diboride were studied in a broad temperature range (1000–1600SC) using X-ray powder diffraction analysis and SEM/EDS at different accelerating voltages, and TOF-ND analysis. It was stated that the phase composition of the products varies depending on the experimental conditions and can include the intermetallic W_x_Ir_1−x_ phases, IrB_1.1_, and ternary W–Ir–B borides. A set of analytical techniques unambiguously confirmed that the novel ternary boride with statistical composition W_2.2±0.1_Ir_5_B_2.3±0.2_ was formed together with the known W_2_Ir_3_B_6−x_ ternary boride at temperatures above 1300 °C. No phase with a similar composition (W_2_Ir_5_B_2_) has been previously reported for the W–Ir–B system. It was suggested that emergence of liquid iridium boride at temperatures above 1300 °C improves mass transfer and contributes to the formation of a variety of ternary borides, W_2_Ir_3_B_6_ and W_2_Ir_5_B_2_. The Vickers microhardness values for new ternary W_2_Ir_5_B_2_ boride were measured at a load of 0.245 N. The microhardness was found to be equal to 15.5 ± 3.1 GPa. Based on these findings, the new ternary boride can be considered a hard phase.

Due to compositional diversity, the phases formed via the reaction between tungsten boride and iridium can have a broad range of physical and chemical properties. This fact offers additional opportunities for designing structural materials with improved properties for high-temperature applications.

## Figures and Tables

**Table 1:** The molar ratios of tungsten boride and iridium powder.

Mixture	W_2_B (mol.%)	WB (mol.%)	Ir (mol.%)	Ir:W:B Molar Ratio
1:1	26.9	9.6	63.5	1:1:0.6
3:1	11.9	4.2	83.9	3:1:0.6

**Table 2:** The quantitative and qualitative TOF-ND analysis data for the product obtained at 1600 °C for 4 h.

Phase	Crystal Structure	Lattice Parameters, Å	Volume, Å^3^	Content, wt.%
WB	I 4_1_/amd	a = 3.113 c = 16.90	163.8	16
WIr_3_	P 6_3_/mmc	a = 5.499 c = 4.394	115.07	19
WIr	P mma	a = 4.417 b = 2.760 c = 4.808	58.62	23
W_x_Ir_1−x_	P 6_3_/mmc	a = 2.771 c = 4.457	29.65	42

**Table 3:** Comparison of the mean Vickers microhardness values (load, 0.245 N) for different intermetallic W–Ir phases studied in this work to those reported previously.

Reference	Microhardness, GPa	
	W, ~26 at.%	W, ~36 at.%
This study	10.7 ± 1.6	13.1 ± 2.0
Tylkina et al. [18]	10.8	12.7
Raub et al. [15]	13.2 *	15.2 **

* 24 at.% W; ** 34 at.% W.

## Data Availability

The data presented in this study are available on request from the corresponding author.

## Supplementary Materials

The following supporting information can be downloaded at: https://www.mdpi.com/article/10.3390/ma15217522/s1, Figure S1: SEM image of the sample heat-treated at 1300 °C after preparation of cross-section; Figure S2: The TOF-ND of the products obtained in the 3:1 mixture at 1600 °C. The *d* range in which the unidentified peaks were detected is denoted by dashed line; Figure S3: The BSE SEM/EDS image and elemental composition of the area belonging to the W_x_Ir_1−x_ intermetallic phase (where x ≈ 0.33); Figure S4. The BSE SEM/EDS image and elemental composition of the area belonging to the W_x_Ir_1−x_ intermetallic phase (where x ≈ 0.33); Table S1: The unidentified *d* spacing and the corresponding 2Θ. Reference [43] are cited in the supplementary materials.
