# Supplementary material for: Evolution of the Microstructure and Phase Composition of the Products Formed in the Reaction between Iridium and W2B
Source: Materials (Basel). 2022 Oct 26;15(21):7522. doi: 10.3390/ma15217522 (PMC9653812; doi:10.3390/ma15217522)
Supplement: Supplementary file 1 [file materials-15-07522-s001.zip › materials-1952857-supplementary.pdf]

SUPPLEMENT

Evolution of the microstructure and phase composition of the products formed in the reaction between iridium and W_2B

D.A. Bannykh[1)], V.V. Lozanov[1)], T.A. Gavrilova[2)], A.I. Beskrovny[3)], N.I. Baklanova[1)]

[1)] *Institute of Solid State Chemistry and Mechanochemistry SB RAS, 18 Kutateladze Str., Novosibirsk 630090, Russia*

[2]*Rzhanov Institute of Semiconductor Physics SB RAS, 13 Lavrentiev Ave., Novosibirsk 630090, Russia*

[3]*Joint Institute for Nuclear Research, 6 Joliot-Curie Str., Dubna 141980, Russia*

1. Experimental

The XRD measurements were calibrated using Al_2O_3 as a standard (NIST 1976a). Quantitative phase analysis and lattice parameter refinement were performed using the Rietveld method (Topas 4.2 software, Bruker AXS, Germany). The phases analyzed were cross-referenced against the Inorganic Crystal Structure Database ICSD (FIZ Karlsruhe, Germany, 1996). The crystal structure data for WB, WIr, WIr_3 and W_xIr_{1-x} solid solution were referenced after Springer Materials database.

The neutron flux was $5 \cdot 10^6 \, {}^{nu}/_{cm^2 \cdot s}$, and the neutron flight path length was 24.45 m [1]. Because of the large cross sections of absorption for boron and iridium ($\sigma_{abs}(B) = 767$ barn, $\sigma_{abs}(Ir) = 425$ barn), the spectrum was accumulated for 12 h. Spectra were measured at $\theta = 85.8°$ (neutron backscattering). The resolution was $\Delta d/d \approx 1\%$; the range of neutron wavelengths was ∼1−12 Å with the maximum intensity at ∼1.9 Å. The TOF-ND patterns were fitted using the GSAS-II software[2].

The morphology and elemental composition were studied using a Hitachi TM 1000 scanning electron microscope coupled with a SwiftED™ energy dispersive X-ray spectroscopy (EDS) detector (Oxford Instruments Analytical Ltd., UK, accelerating voltage of 15 kV).

Additionally, the SEM/EDS studies were performed with a Mira 3 LMU high-resolution scanning electron microscope (TESCAN) equipped with an INCA Energy 450 XMax 80 EDS detector with accelerating voltage of 20 kV. Cross-sections of the samples were prepared for in-depth analysis of the microstructure and local elemental composition of the powdered products. The samples were embedded into epoxy resin, cut with a diamond saw, and carefully polished using a set of polycrystalline diamond suspensions with particle size ranging from 9 to 1 μm (Monosyn Duo, Synercon, Germany). The probe current and the energy shift of the recorded spectrum were calibrated using metallic cobalt as a standard. A SU8220 scanning electron microscope (Hitachi, Japan) equipped with QUAD and Quantax 60 EDS detectors (Bruker, USA) was used for detecting boron (K-line) together with tungsten (M-series) and iridium (M-series) at an accelerating voltage of 6 kV (shared-use facilities of the “Nanostructures” Center, Institute of Semiconductor Physics, SB RAS, Novosibirsk) [3,4]. Low accelerating voltage provides a sufficient peak to ground ratio for quantification of boron [3]. In accordance with Bruker recommendation, Ni_3B was used as a calibration standard. SEM images were processed using the ImageJ software (National Institute of Health) to evaluate the sizes of product particles and aggregates.

2. RESULTS

2.1. The 1 : 1 mixtures

Scanning electron microscopy

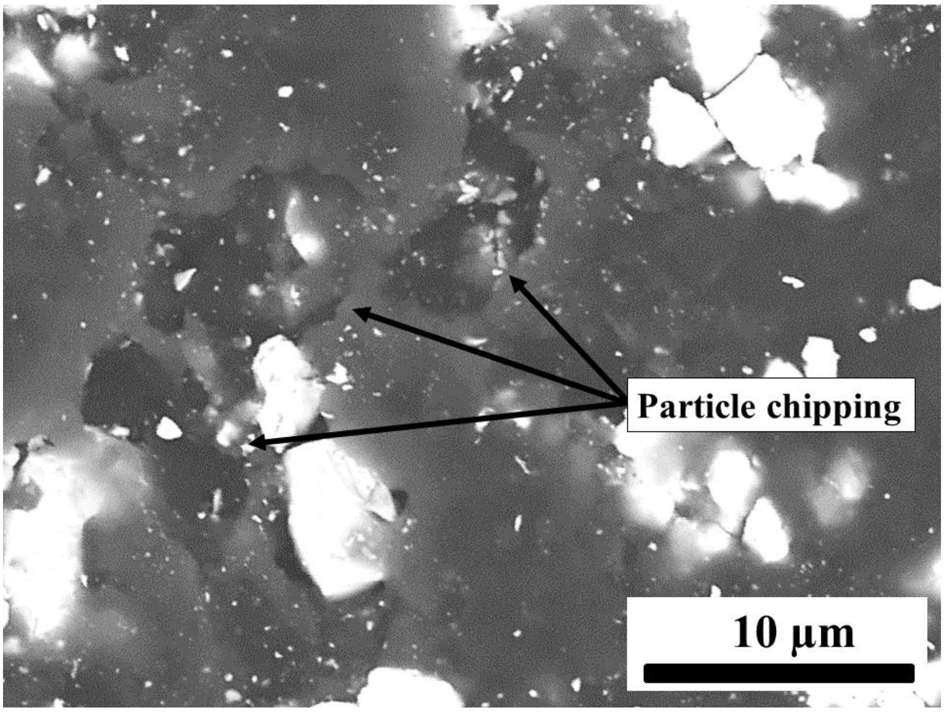

Figure S1. SEM image of the sample heat-treated at 1300°C after preparation of cross-section

2.2. The 3 : 1 mixtures

Phase composition

X-Ray diffraction analysis

Table S1. The unidentified *d* spacing and the corresponding 2Θ

2Θ	23.11	26.67	30.64	31.77	35.78	44.47	49.27	50.48	51.18
D, Ȧ	3.84	3.34	2.91	2.81	2.50	2.03	1.84	1.80	1.78

The TOF-ND analysis

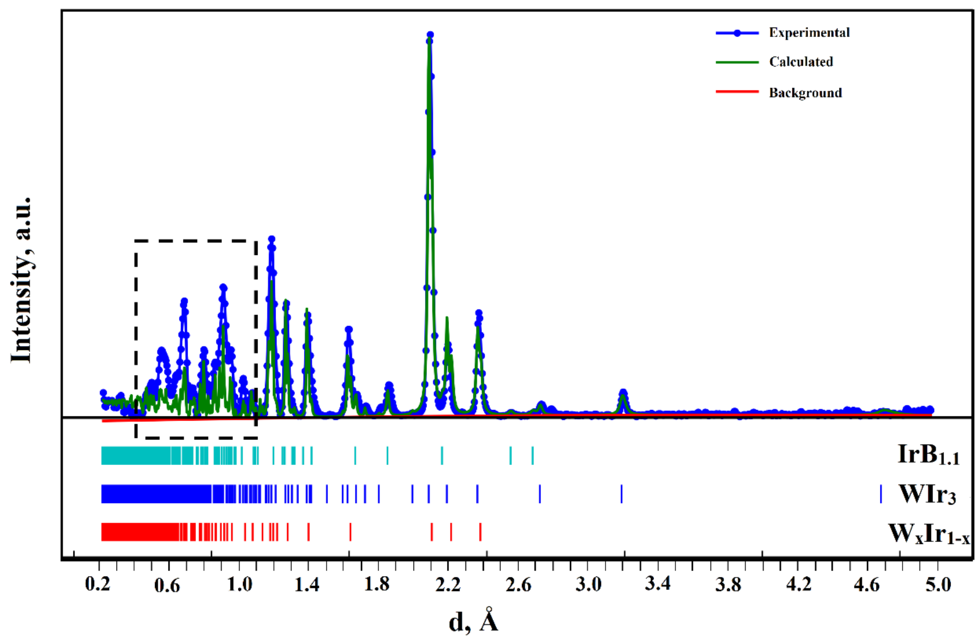

Figure S2. The TOF-ND of the products obtained in the 3 : 1 mixture at 1600°C. The *d* range in which the unidentified peaks were detected is denoted by dashed line.

Elemental composition

The BSE SEM/EDS results, accelerating voltage of 6 kV

Spectrum: WIr 8

Element	Series	unn. C [wt.%]	norm. C [wt.%]	Atom. C [at.%]	Error (3 Sigma) [wt.%]
Boron	K-series	0.00	0.00	0.00	0.00
Tungsten	M-series	25.23	30.41	31.36	3.36
Iridium	M-series	57.74	69.59	68.64	7.39
	Total:	82.96	100.00	100.00	

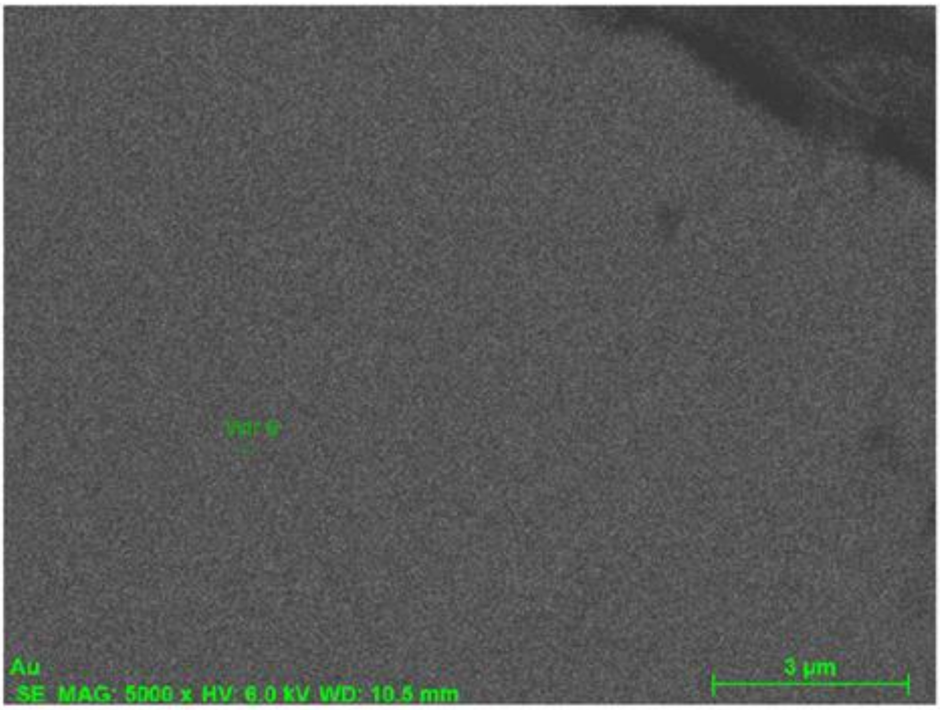

Figure S3. The BSE SEM/EDS image and elemental composition of the area belonging to the W_xIr_{1-x} intermetallic phase (where $x \approx 0.33$)

Spectrum: WIr 1

Element	Series	unn. C [wt.%]	norm. C [wt.%]	Atom. C [at.%]	Error (3 Sigma) [wt.%]
Boron	K-series	0.00	0.00	0.00	0.00
Tungsten	M-series	22.96	31.02	31.98	3.07
Iridium	M-series	51.05	68.98	68.02	6.56
	Total:	74.01	100.00	100.00	

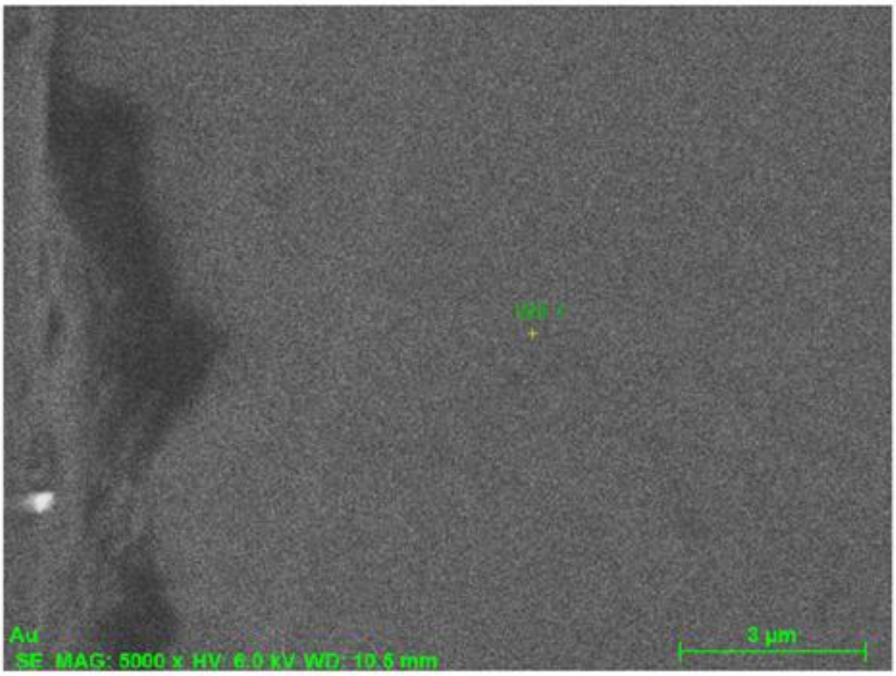

Page 2 / 40

Figure S4. The BSE SEM/EDS image and elemental composition of the area belonging to the W_xIr_{1-x} intermetallic phase (where $x \approx 0.33$)

References:

[1] A.M. Balagurov, A.I. Beskrovnyy, V.V. Zhuravlev, G.M. Mironova, I.A. Bobrikov, D. Neov, S.G. Sheverev, Neutron diffractometer for real-time studies of transient processes at the IBR-2 pulsed reactor, J. Synch. Investig. 10 (2016) 467–479. https://doi.org/10.1134/S1027451016030046.

[2] B.H. Toby, R.B. Von Dreele, *GSAS-II*: the genesis of a modern open-source all purpose crystallography software package, J Appl Crystallogr. 46 (2013) 544–549. https://doi.org/10.1107/S0021889813003531.

[3] J. Berlin, Analysis of boron with energy dispersive X-ray spectrometry, Imaging & Microscopy. 13 (2011), 19–21. https://www.yumpu.com/en/document/read/33185105/analysis-of-boron-with-energy-dispersive-x-ray-spectrometry-bruker

[4] J. Ruiz-Vargas, N. Siredey-Schwaller, P. Noyrez, S. Mathieu, P. Bocher, N. Gey, Potential and limitations of microanalysis SEM techniques to characterize borides in brazed Ni-based superalloys, Materials Characterization. 94 (2014) 46–57. https://doi.org/10.1016/j.matchar.2014.04.009.
